# Variants of *PCSK9* Gene Are Associated with Subclinical Atherosclerosis and Cardiometabolic Parameters in Mexicans. The GEA Project

**DOI:** 10.3390/diagnostics11050774

**Published:** 2021-04-26

**Authors:** Erasmo Zamarrón-Licona, José Manuel Rodríguez-Pérez, Rosalinda Posadas-Sánchez, Gilberto Vargas-Alarcón, Manuel Alfonso Baños-González, Verónica Marusa Borgonio-Cuadra, Nonanzit Pérez-Hernández

**Affiliations:** 1 Departamento de Biología Molecular, Instituto Nacional de Cardiología Ignacio Chávez, Ciudad de México 14080, Mexico; erasmo.zamarron@cbtis32.edu.mx (E.Z.-L.); josemanuel_rodriguezperez@yahoo.com.mx (J.M.R.-P.); gvargas63@yahoo.com (G.V.-A.); 2 Departamento de Endocrinología, Instituto Nacional de Cardiología Ignacio Chávez, Ciudad de México 14080, Mexico; rossy_posadas_s@yahoo.it; 3 Centro de Investigación y Posgrado, División Académica de Ciencias de la Salud, Universidad Juárez Autónoma de Tabasco, Villahermosa 86150, Mexico; manuel_banos@hotmail.com; 4 Departamento de Genética, Instituto Nacional de Rehabilitación Luis Guillermo Ibarra, Ciudad de México 14389, Mexico; vborgoni@yahoo.com.mx

**Keywords:** subclinical atherosclerosis, proprotein convertase subtilisin/kexin type 9, vascular diseases, coronary artery calcium, genetic association, polymorphisms

## Abstract

Background: Coronary artery disease (CAD) is a chronic, inflammatory, and complex disease associated with vascular risk factors. Nowadays, the coronary artery calcium (CAC) is a specific marker of the presence and extent of atherosclerosis. Additionally, CAC is a predictor of future coronary events in asymptomatic individuals diagnosed with subclinical atherosclerosis (CAC > 0). In this study, our aim is to evaluate the participation of two polymorphisms of the *PCSK9* gene as genetic markers for developing subclinical atherosclerosis and cardiometabolic risk factors in asymptomatic individuals. Methods: We analyzed two PCSK9 polymorphisms (rs2479409 and rs615563) in 394 individuals with subclinical atherosclerosis and 1102 healthy controls using real time- polymerase chain reaction (PCR). Results: Under various inheritance models adjusted for different confounding factors, the rs2479409 polymorphism was associated with an increased risk of developing subclinical atherosclerosis (OR = 1.53, P recessive = 0.041). Both polymorphisms were significantly associated with several cardiometabolic parameters. Conclusions: Our data suggest that rs2479409 polymorphism could be envisaged as a risk marker for subclinical atherosclerosis.

## 1. Introduction

Coronary artery disease (CAD) is a chronic, inflammatory, and complex disease, and it is considered the main cause of death worldwide. The mechanisms involved in the development of this pathology include cell proliferation in arterial walls as a result of the release and activity of cytokines, growth factors, thrombotic, hemostatic, and immunological molecules. The consequence of cell proliferation in arterial walls is the formation of atheromatous plaques that can calcify; therefore, coronary artery calcium (CAC) is a specific marker of the presence and extent of atherosclerosis. CAC is also a predictor of future coronary events in asymptomatic individuals who have been diagnosed with subclinical atherosclerosis (SA) (CAC > 0) [1,2].

Nowadays, the presence of SA is considered as an independent marker of cardiovascular risk, even more than traditional risk factors such as obesity, hypertension, smoking habit, hyperlipidemia, and a family history of CAD [3].

Additionally, epigenetic and genetic factors are considered to influence the development of CAD [2,4,5,6]. In this sense, genetic research of human diseases has helped to identify gene variants with functional implications over several biological signaling pathways related to the vascular health process. Among these pathways, the proprotein convertase subtilisin/kexin type 9 (PCSK9) is a key molecule which is synthesized and secreted mainly by hepatocytes, but with lower expression in the brain, kidney and intestine. This molecule has an important role in low-density lipoprotein cholesterol (LDL-C) homeostasis, binding to the LDL receptor (LDLR) to stimulate its lysosomal degradation in hepatocytes [7,8,9]. The human *PCSK9* gene is located on chromosome 1p32.3 and contains 12 exons. This gene is polymorphic and some of its variants have been associated with coronary disease [10].

Up to now, there is only one study that has indicated an association of *PCSK9* gene polymorphisms with SA [11]. However, associations of *PCSK9* gene polymorphisms with CAD and comorbidities, such as type 2 diabetes mellitus, have been reported in different populations with inconsistent results [8,12,13,14,15].

We consider that the identification of genetic markers in early stages of CAD will help prevent or retard its progression and thus avoid later complications. As there are no previous studies exploring the association of *PCSK9* polymorphisms with SA in the Mexican population, our aim was to analyze the association of rs2479409 and rs615563 polymorphisms of the *PCSK9* gene with subclinical atherosclerosis in a well characterized cohort from clinical, biochemical, and tomographic points of view. Additionally, we also aimed to analyze the association of these two polymorphisms with cardiometabolic parameters and perform an in silico analysis of these polymorphic sites.

For this study, polymorphisms were selected, considering previous association reports on different vascular diseases, that present a minor allele frequency (MAF) greater than the 20% reported in 1000 Genomes Project and also, based on the results from an in silico analysis to predict a possible functional role.

## 2. Materials and Methods

### 2.1. Study Population

This study was approved by the Research and Ethics Committee of the “Instituto Nacional de Cardiología Ignacio Chávez” (INCICh—Registry number 15–915) and complies with the Declaration of Helsinki. All the participants signed an informed consent.

The present study included a total of 1496 participants who were asymptomatic, apparently healthy, and without a personal or family history of CAD. The participants were recruited from blood banks and by invitation through brochures posted at the institute. These participants belong to the GEA Mexican Cohort. All participants underwent a computed tomography (CT) in the abdomen and chest using a multidetector computed tomography system (Somatom Sensation, Germany). Scans were interpreted to evaluate and quantify the CAC score through the Agatston method [16]. After the CT, 394 individuals were assorted in the group of SA (participants with CAC score greater than zero), whereas 1102 individuals formed the control group (participants with CAC score of zero). The exclusion criteria for both study groups were heart failure, renal failure, liver disease, oncological diseases, thyroid disease, and premature atherosclerosis. Clinical, demographic, anthropometric, biochemical, and metabolic parameters, as well as cardiometabolic risk factors, were assessed similarly to previous reports [17,18,19,20]. Body mass index (BMI) was obtained as weight [kg]/height [m^2^]. Central obesity was considered when women presented 80 cm (or more) of waist circumference and men presented 90 cm (or more) of waist circumference [21]. Smoking habits were defined when participants self-reported the current use of tobacco. Hypertension was considered as high values of systolic blood pressure and diastolic blood pressure (≥140 mmHg and ≥90 mmHg, respectively) or by reporting the use of oral antihypertensives. Type 2 diabetes mellitus (T2DM) was defined following the American Diabetes Association (ADA) criteria, with a fasting glucose ≥ 126 mg/dL concentrations, and was also considered when individuals reported the use of hypoglycemic agents. Hypertriglyceridemia and increased low-density lipoprotein-cholesterol (LDL-C), as well as metabolic syndrome were defined according to the American Heart Association, National Heart, Lung, and Blood Institute Scientific Statement of the Metabolic Syndrome [22]. Insulin resistance was estimated through the homeostasis model assessment (HOMA-IR), considered as present when values were ≥75th percentile (3.38 in men and 3.66 in women). Hyperinsulinemia was established when insulin concentrations were ≥75th percentile (15.20 μIU/mL in men and 16.97 μIU/mL in women). Increased alkaline phosphatase was considered when values were ≥75th percentile (90.25 IU/L in women and 83.0 IU/L in men), increased gamma-glutamyltranspeptidase was considered when concentrations were ≥75th percentile (28.0 IU/L in women and 34.0 IU/L in men), increased interleukin1β was defined when values were ≥75th percentile (0.19 IU/L in women and 0.28 IU/L in men), and decreased interleukin-10 was considered when concentrations were ≤25th percentile (0.30 IU/L in women and 0.27 IU/L in men). These cut-off points were decided by taking into consideration data from the GEA cohort sample of 185 women and 131 men without obesity and normal values of lipids, fasting glucose, and blood pressure.

All participants self-reported having Mexican ancestry (of at least three generations). In addition, we previously determined the genetic background of this population, using 265 ancestry informative markers, and the results showed that all individuals in our sample (with and without SA) had a similar genetic background; therefore, there was no genetic bias in the present study [23].

### 2.2. Genetic Determination

High-quality genomic DNA was extracted from peripheral blood samples using commercial kits (QIAamp DNA Blood Mini kit, Qiagen, Germany). Two polymorphisms were analyzed in the study (rs2479409—assay: C___2018190_10 with context sequence [VIC/FAM]:AGAATTCTGAATGTACCTATATGAC[A/G]TCTTTGCAAACTTAAAACCTGAATC, and rs615563—assay: C___3184713_10 with context sequence [VIC/FAM]: ATCACGCTCCCCTTTGGAAGTGCTC[A/G]GCCGATGAGCTCACAGGCACATGTC. The polymorphisms were determined using 5′ exonuclease TAQMAN genotyping assays on 7900HT-Real Time equipment, using a discrimination allelic software (Applied Biosystems, Foster City, CA, USA). To validate the correct assignment of genotypes, ten percent of samples in both groups were determined in duplicate and the results showed 100% concordance.

### 2.3. In Silico Analysis

The possible functional effect of the studied polymorphisms was evaluated using SNPinfo Web Server, a bioinformatics tool that identifies transcription factors binding sites produced by changes in specific polymorphic sites [24].

### 2.4. Statistical Analysis

The two polymorphisms analyzed were in the Hardy–Weinberg equilibrium. Data are shown as medians and interquartile ranges, as well as medias and standard deviations or frequencies and percentages, as required. The continuous and categorical variables in both groups were analyzed with Student’s t test, Mann–Whitney U test, Kruskal–Wallis test, and chi-square test, as required. The association of rs2479409 and rs615563 *PCSK9* polymorphisms with SA was evaluated using a logistic regression through different inheritance models: additive (major allele homozygotes vs. heterozygotes vs. minor allele homozygotes), dominant (major allele homozygotes vs. heterozygotes + minor allele homozygotes), recessive (major allele homozygotes + heterozygotes vs. minor allele homozygotes), heterozygote (heterozygotes vs. major allele homozygotes + minor allele homozygotes), codominant 1 (major allele homozygotes vs. heterozygotes), and codominant 2 (major allele homozygotes vs. minor allele homozygotes). The models were adjusted by age, gender, body mass index (BMI), smoking habits, concentrations of LDL-cholesterol, and type 2 diabetes mellitus (T2DM). To assess the associations between the two polymorphisms and cardiometabolic risk factors in both groups, models were adjusted by age, gender, and BMI. All analyses were performed using SPSS (statistical package for the social sciences, v24.0). In this study, a *p*-value of <0.05 was considered statistically significant.

The patterns of linkage disequilibrium (LD) and construction of haplotypes were performed using the Haploview Software (Haploview v4.1-Broad Institute of Massachusetts, Cambridge, MA, USA) [25].

## 3. Results

### 3.1. Assessment of Metabolic, Clinical, and Cardiovascular Risk Factors

Metabolic and clinical parameters of the population studied are depicted in Table 1. In comparison to the control group, individuals with SA showed increased values of diastolic and systolic blood pressure, waist circumference, low-density lipoprotein cholesterol (LDL-C), glucose, homeostasis model assessment insulin resistance (HOMA), and gamma-glutamyltranspeptidase. We also observed that the prevalence of T2DM, insulin resistance, and metabolic syndrome was higher in individuals with SA when compared to the control group, as shown in Table 2.

### 3.2. Association of rs615563 and rs2479409 Polymorphisms and Haplotypes with SA

**Figure 1:**
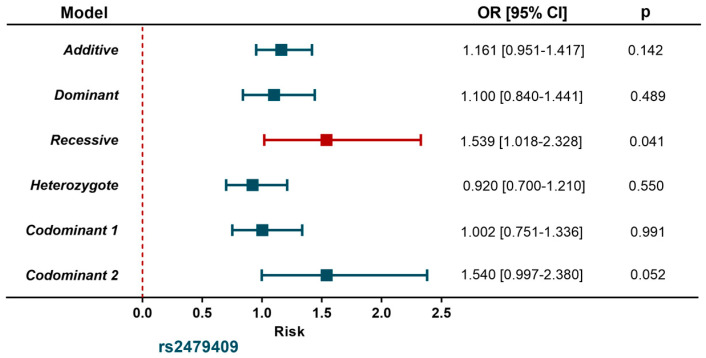
Association between *PCSK9* gene polymorphism and subclinical atherosclerosis.

The distribution of rs615563 polymorphism was similar in individuals with SA and the control group. Conversely, in the recessive model adjusted for age, gender, BMI, smoking habits, LDL-C and T2DM, the rs2479409 *A* allele was associated with a high risk of SA (OR = 1.539, 95% CI = 1.018–2.328, *p* = 0.041) (Figure 1).

Controls (n = 1102); Subclinical atherosclerosis (n = 394). Models were adjusted for age, sex, body mass index, smoking habits, LDL-cholesterol, type 2 diabetes mellitus.

Moreover, none of the polymorphisms showed linkage disequilibrium evidence (D’ = 0.57) and none of the four haplotypes constructed were associated with SA. These data are depicted in Table 3.

### 3.3. Association of rs615563 and rs2479409 Polymorphisms with Low-Density Lipoprotein Cholesterol and Triglycerides Concentrations and Cardiometabolic Parameters

Concentrations of LDL-C and triglycerides (TG) were determined in the whole sample (individuals with SA and controls). Then, the concentrations of LDL-C and TG were analyzed by stratifying the different genotypes for both polymorphisms in the study population, the results were similar for the rs615563. However, different TG concentrations were observed in the rs2479409 genotypes in the control group (*p* = 0.016) (Table 4).

The association of rs615563 and rs2479409 with cardiometabolic parameters was evaluated independently in individuals with SA and controls. In individuals with SA and under different models, the rs2479409 polymorphism was associated with a low risk of insulin resistance of adipose tissue (OR = 0.426, 95% CI = 0.218–0.829 P codominant 1 = 0.012), and a low risk of having increased interleukin 1β > p75 (OR = 0.397, 95% CI = 0.176–0.893, P recessive = 0.026) (Table 5).

The rs615563 polymorphism was significantly associated with a decreased risk of central obesity (OR = 0.093, 95% CI = 0.010–0.839, P recessive = 0.034), metabolic syndrome (OR = 0.593, 95% CI = 0.388–0.907, P additive = 0.016), hyperinsulinemia (OR = 0.556, 95% CI = 0.363–0.881, P additive = 0.012; OR = 0.509, 95% CI = 0.300–0.863, P codominant 1 = 0.012), insulin resistance (OR = 0.486, 95% CI = 0.286–0.829, P codominant 1 = 0.012), insulin resistance of adipose tissue (OR = 0.453, 95% CI = 0.453–0.754, P codominant 1 = 0.002), low risk of increased alkaline phosphatase > p75 (OR = 0.636, 95% CI = 0.421–0.962, P dominant = 0.032), and low risk of having decreased interleukin 10 < p25 (OR = 0.487, 95% CI = 0.264–0.896, P codominant 1 = 0.021). These data are represented in Table 5.

With regards to metabolic abnormalities in the control group, the rs2479409 polymorphism was statistically associated with a decreased risk of metabolic syndrome (OR = 0.768, 95% CI = 0.593–0.994, P dominant = 0.045), low risk of having increased alkaline phosphatase > p75 (OR = 0.758, 95% CI = 0.625–0.919, P additive = 0.005; OR = 0.703, 95% CI = 0.548–0.903, P dominant = 0.006; OR = 0.729, 95% CI = 0.560–0.950, P codominant 1 = 0.019; OR = 0.603, 95% CI = 0.386–0.943, P codominant 2 = 0.027), and low risk of increased gamma-glutamyltranspeptidase (OR = 0.760, 95% CI = 0.588–0.982, P heterozygote = 0.036; OR = 0.751, 95% CI = 0.575–0.981, P codominant 1 = 0.036). While the rs615563 polymorphism was associated with a lower risk of hypertriglyceridemia (OR = 0.358, 95% CI = 0.149–0.862, P recessive = 0.022; OR = 0.374, 95% CI = 0.155–0.903, P codominant 2 = 0.029), and low risk of having increased interleukin 1β > p75 (OR = 0.767, 95% CI = 0.598–0.985, P additive = 0.037). These findings are depicted in Table 6. All inheritance models were adjusted for age, gender, and BMI.

## 4. Discussion

Lately, the study of a genetic component in subclinical atherosclerosis has taken clinical relevance in public health worldwide. The detection of risk genes associated with SA could improve the understanding of its physiopathogenesis. Therefore, in the present study, we analyzed two polymorphisms (rs2479409 and rs615563) of *PCSK9* gene, in 1102 controls and 394 individuals with SA, in order to determine a possible risk of developing SA in a Mexican population. Moreover, we investigated the association of these two polymorphisms with cardiometabolic risk factors.

A dissimilar distribution of rs2479409 was observed in individuals with SA when compared with controls. A larger presence of the *A* allele in SA suggests that it could be a risk marker for Mexican individuals with SA. On the other hand, high serum levels of PCSK9 have been associated with different vascular diseases such as vasculitis, atherosclerosis, arterial calcification, cerebrovascular, and aortic diseases [26]. Additionally, various reports have linked PCSK9 with systemic lupus erythematosus [27,28], rheumatoid arthritis [29,30], psoriasis [31,32], systemic sclerosis [33], and nephrotic syndrome [34].

Mostaza et al. analyzed the association of rs11591147 polymorphism with lipid levels and subclinical vascular disease in 1188 individuals free of cardiovascular disease. They found a significant association with SA; however, no association with lipid levels was found [11]. Recently, Qiu et al. performed a meta-analysis of rs505151 and rs11591147 polymorphisms of *PCSK9* and found an association of these polymorphisms with cardiovascular risk and high lipid levels [6]. On the other hand, Reddy et al. reported an association of rs505151 polymorphism with CAD in patients from north India [12]. Although the associations were with different polymorphisms, if we consider that it is the same gene, then these results agree with our findings. Moreover, different polymorphisms of the *PCSK9* gene influence circulating levels of molecules related to the lipid metabolism that lead to a risk for dyslipidemias and cardiovascular diseases. It is known that the rs2479409 polymorphism has been associated with LDL-C and the rs615563 polymorphism is implicated with TG circulating levels. In our study, when analyzing the association between both polymorphisms and LDL-C and TG concentrations, the rs2479409 polymorphism showed an associated with TG levels in the control group. However, currently, there is controversy in the results with positive and negative associations. Luo et al. observed an association with risk for rs2479409 for TC (total-cholesterol) in the Chinese population [13]. Kulminski et al. found that the rs2479409 showed a protective role with TC levels [35]. Small et al. reported an association of the rs2479409 with decreased LDL-C levels in patients with CAD and ischemic stroke (IS) [36]. Harrison et al. did not find an association with the rs2479409 and LDL-C circulating levels [37]. Guo et al. observed in the rs615563 an association with the risk for TG circulating levels [38], and Guo et al. detected in the rs615563 an association for a decreased risk of TC and TG circulating levels in the Chinese population [14].

On the other hand, the rs615563 polymorphism in the control group was associated with a low risk of developing hypertriglyceridemia. These differences between these studies and ours could be due to the inclusion criteria used in each study. Luo et al. and Guo et al. [38] evaluated patients with dyslipidemia; Kulminski et al. re-analyzed previous associations in a GWAS meta-analysis; Small et al. included coronary patients; Harrison et al. selected patients with abdominal aortic aneurism; Guo et al. [14] studied healthy individuals in two different populations from China, whereas in our study only individuals with SA, considering the CAC > 0 score, were included. Furthermore, all individuals we included were asymptomatic and had no family history nor personal history of CAD. Thus, participants in our study were different from those studied by the previously mentioned authors.

In addition, studies in populations have identified different mutations in the *PCSK9* gene that are implicated in changes in circulating LDL cholesterol levels. Gain of function (GOF) mutations reduce LDLR levels in the liver, resulting in increased LDL-C circulating levels, leading to a high risk of developing CAD; whereas loss of function (LOF) mutations increase LDLR levels, lowering LDL-C circulation levels and, therefore, it has a protective effect against CAD [39,40,41,42,43,44]. Therefore, the above evidence suggests the complexity involved in the regulation of circulating levels of molecules implicated in the lipid metabolism by the activity of the *PCSK9* gene.

Next, we explored the association of rs2479409 and rs615563 polymorphisms with metabolic abnormalities by comparing healthy controls to individuals with SA. In the control group, the rs2479409 was associated with a lower risk of metabolic syndrome, a low risk of having increased alkaline phosphatase >p75, and a low risk of increased gamma-glutamyltranspeptidase. Several reports have shown that metabolic syndrome increases the risk of cardiovascular events [45,46,47]. Recent studies have suggested that an increased serum alkaline phosphatase is a predictor of cardiovascular disease and vascular calcification [48,49]. In the same way, some studies have associated high serum levels of gamma-glutamyltranspeptidase with a high prevalence of CAD and cardiovascular risk factors [50,51,52].

The rs615563 polymorphism was associated with a decreased risk of hypertriglyceridemia and a low risk of having high levels of interleukin 1β > p75. It is well known that increased serum levels of triglycerides are an important cardiovascular risk factor, and have also been associated with atherosclerosis [53,54,55]. Finally, high levels of interleukin 1β are involved in inflammation and atherogenesis [56,57].

We also observed that in the group of individuals with SA, the rs2479409 polymorphism was associated with a low risk of having insulin resistance of adipose tissue and a low risk of increased interleukin 1β > p75. The rs615563, on the other hand, was associated with a lower risk of having central obesity, metabolic syndrome, hyperinsulinemia, insulin resistance, insulin resistance of adipose tissue, low risk of increased alkaline phosphatase >p75, and low risk of having decreased interleukin 10 <p25.

It is important to genetically characterize individuals with SA in order to establish an early and timely detection and to prevent cardiovascular disease [58]. In our study, none of the *PCSK9* haplotypes were associated with SA. Up to now, there is no evidence of haplotypes associated with SA; thus, additional studies are required to understand the true role of *PSCK9* gene polymorphisms in SA.

The bioinformatics analysis showed that changes in the rs2479409 polymorphism produced binding sites for AP-1, YY1, and HOXA9 transcription factors, all of them related to vascular disease. AP-1 is a known family of dimeric complexes implicated in different cellular processes such as differentiation, cell proliferation, survival, and death, and it has been involved in different diseases, in particular those of inflammatory etiology [59,60]. In multifactorial pathologies, modifications of AP-1 binding sites have been reported, and those sites are related to the epigenetic regulation and genetic risk of coronary artery disease [61].

YY1 is a zinc finger nuclear that can activate or repress and initiate transcription according to structure and environment conditions. In vascular diseases, YY1 can act as a repressor of several gene promotors involved in atherogenesis such as hormones, grown factors, and cytokines. Santiago et al. demonstrated that YY1 is differentially expressed in healthy and damaged human arteries [62]. HOXA9 is a transcription factor with important implications in the expression of several genes related to endothelial biology, including VE-cadherin, *VEGFR-2*, eNOS [63]. It is well known that endothelial dysfunction represents an initial step for progression to cardiovascular disease [64].

The present study has important strengths: we included a large cohort of Mexican individuals ethnically homogenous to ensure that the population stratification was not biased. In the same way, in all the participants, it was possible to obtain information on clinical, demographic, tomographic, and biochemical parameters. Nonetheless, some limitations should also be recognized: our results have to be interpreted with caution, considering that the participants were not randomly selected. Furthermore, our results were generated from individuals belonging to the basal phase of GEA Mexican study with a cross-sectional design.

## 5. Conclusions

In conclusion, our results suggest an association of *PCSK9* rs2479409 polymorphism with subclinical atherosclerosis. Moreover, both *PCSK9* polymorphisms (rs2479409 and rs615563) were associated with various biochemical parameters in individuals with SA and controls. As far as we know, this is the first research that has detected these associations. Nevertheless, additional studies in other ethnicities are required to confirm these findings.

## Figures and Tables

**Table 1:** Clinical and metabolic characteristics of the study groups.

Characteristics	Control (N = 1102)	Subclinical Atherosclerosis (N = 394)	* p
Age (years)	51 ± 9	59 ± 8	<0.001
Gender (% male)	41	75.4	<0.001
Waist circumference (cm)	93.6 ± 11.2	97.2 ± 10.7	<0.001
Body mass index (kg/m^2^)	27.9 [25.4–30.8]	28.1 [25.9–31.0]	0.080
Systolic blood pressure (mmHg)	112 [104–125]	122 [111–134]	<0.001
Diastolic Blood pressure (mmHg)	71 [65–76]	75 [69–82]	<0.001
Low-density lipoprotein cholesterol(mg/dL)	115 [95–134]	124 [102–145]	<0.001
Glucose (mg/dL)	90 [84–97]	94 [86–105]	<0.001
Insulin (µUI/mL)	16.9 [12.4–23.1]	18 [12.7–24.5]	0.071
Homeostasis model assessment insulin resistance (HOMA)	3.8 [2.6–5.5]	4.4 [2.9–6.8]	<0.001
Insulin resistance of adipose tissue	9.3 [6.1–14.3]	10.3 [6.6–14.4]	0.090
Alkaline phosphatase (IU/L)	81 [68–96]	77 [64–92]	0.005
Gamma-glutamyltranspeptidase (IU/L)	26 [18–42]	30 [21–42]	<0.001
Interleukin 1β (pg/mL)	0.18 [0.09–0.29]	0.17 [0.11–0.29]	0.577
Interleukin-10 (pg/mL)	0.44 [0.24–0.99]	0.46 [0.24–1.07]	0.737
Coronary artery calcium (Agatston Units)	0	23.7 [4.3–89.3]	<0.001

Data are shown as mean ± standard deviation or median [interquartile range]. * Student’s *t* test or Mann Whitney U test.

**Table 2:** Cardiovascular risk factors prevalence in the population studied.

Characteristics	Control (N = 1102)	Subclinical Atherosclerosis (N = 394)	* p
Abdominal obesity (%)	81.2	83.2	0.403
Type 2 diabetes mellitus (%)	9.7	22.3	<0.001
Hyperinsulinemia (%)	52.3	61.4	0.002
Insulin resistance (%)	54.3	65.2	<0.001
Insulin resistance of adipose tissue (%)	47.7	57.0	0.002
Metabolic syndrome (%)	38.5	53.3	<0.001
Alkaline phosphatase > p75 (%)	37.2	36.8	0.903
Gamma-glutamyltranspeptidase > p75 (%)	41.1	44.4	0.284
Interleukin 1β > p75 (%)	38.2	30.7	0.013
Interleukin-10 < p25 (%)	31.8	30.2	0.598
Current smoking (%)	22.6	21.3	0.622

Data are shown as percentages. * Chi-square test. >p75: >75th percentile; <p25: <25th percentile.

**Table 3:** Haplotype frequencies in individuals with and without subclinical atherosclerosis.

Haplotypes		Subclinical Atherosclerosis		OR [95% CI]	*p*
		Yes	No		
H1	*GG*	0.627	0.642	0.935 [0.790–1.107]	0.4351
H2	*AG*	0.228	0.207	1.138 [0.936–1.384]	0.2028
H3	*AA*	0.107	0.106	1.004 [0.771–1.308]	0.9733
H8	*GA*	0.038	0.045	0.841 [0.555–1.277]	0.3918

OR: odds ratio; CI: confidence interval. The order of the polymorphisms in the haplotype is according to the position in the chromosome (rs2479409 and rs615563).

**Table 4:** Association between *PSCK9* rs2479409 and rs615563 gene polymorphisms and low-density lipoprotein cholesterol and triglycerides concentrations.

**Polymorphism**	**Genotypes**			* ***p***
**rs2479409**	***GG***	***GA***	***AA***	
***Whole sample***				
n	708	623	165	
Low-density lipoprotein cholesterol (mg/dL)	118 [99–137]	118 [95–138]	117 [94–137]	0.467
Triglycerides (mg/dL)	154 [114–208]	142 [107–201]	147 [106–186]	0.063
***Control group***				
n	527	461	114	
Low-density lipoprotein cholesterol (mg/dL)	116 [96–135]	115 [93–133]	113 [92–131]	0.461
Triglycerides (mg/dL)	153 [114–209]	137 [101–193]	140 [106–186]	0.016
***Subclinical atherosclerosis***				
n	181	162	51	
Low-density lipoprotein cholesterol (mg/dL)	125 [106–145]	124 [102–146]	121 [94–146]	0.622
Triglycerides (mg/dL)	158 [116–205]	154 [120–209]	157 [115–193]	0.677
**Polymorphism**		**Genotypes**		* ***p***
**rs615563**	***GG***	***GA***	***AA***	
***Whole sample***				
n	1089	367	40	
Low-density lipoprotein cholesterol (mg/dL)	118 [96–138]	118 [97–136]	112 [102–141]	0.837
Triglycerides (mg/dL)	149 [112–204]	146 [110–203]	124 [101–163]	0.113
***Control group***				
n	799	273	30	
Low-density lipoprotein cholesterol (mg/dL)	116 [95–135]	114 [93–132]	110 [103–134]	0.663
Triglycerides (mg/dL)	146 [108–202]	147 [107–205]	121 [100–147]	0.197
***Subclinical atherosclerosis***				
n	290	94	10	
Low-density lipoprotein cholesterol (mg/dL)	124 [102–145]	125 [106–146]	128 [100–148]	0.928
Triglycerides (mg/dL)	160 [121–211]	146 [116–195]	147 [113–193]	0.115

Data are shown as the median [interquartile range]. * Kruskal–Wallis test.

**Table 5:** Association between *PCSK9* gene polymorphisms and metabolic abnormalities in individuals with subclinical atherosclerosis.

Polymorphism	Genotype Frequency			MAF	Model	OR [95% CI]	*p*
**rs2479409**	***GG***	***GA***	***AA***				
Insulin resistance of Adipose tissue							
No (n = 169)	0.389	0.438	0.173	0.391	Codominant 1	0.426 [0.218–0.829]	0.012
Yes (n = 225)	0.507	0.395	0.098	0.296			
Interleukin 1β > p75							
No (n = 273)	0.452	0.395	0.153	0.352	Recessive	0.397 [0.176–0.893]	0.026
Yes (n = 121)	0.491	0.436	0.073	0.293			
**rs615563**	***GG***	***GA***	***AA***				
Central obesity							
No (n = 66)	0.697	0.227	0.076	0.189	Recessive	0.093 [0.010–0.839]	0.034
Yes (n = 328)	0.744	0.241	0.015	0.136			
Metabolic syndrome							
No (n = 184)	0.679	0.283	0.038	0.179	Additive	0.593 [0.388–0.907]	0.016
Yes (n = 210)	0.786	0.200	0.014	0.114			
Hyperinsulinemia							
No (n = 152)	0.664	0.296	0.039	0.188	Additive	0.556 [0.363–0.881]	0.012
Yes (n = 242)	0.781	0.202	0.017	0.118	Codominant 1	0.509 [0.300–0.863]	0.012
Insulin resistance							
No (n = 137)	0.657	0.299	0.044	0.193	Codominant 1	0.486 [0.286–0.829]	0.012
Yes (n = 257)	0.778	0.206	0.016	0.119			
Insulin resistance of adipose tissue							
No (n = 169)	0.660	0.309	0.031	0183	Codominant 1	0.453 [0.453–0.754]	0.002
Yes (n = 225)	0.786	0.191	0.023	0.113			
Alkaline phosphatase > p75							
No (n = 249)	0.418	0.438	0.145	0.363	Dominant	0.636 [0.421–0.962]	0.032
Yes (n = 145)	0.531	0.366	0.103	0.286			
Interleukin 10 < p25							
No (n = 275)	0.716	0.260	0.024	0.154	Codominant 1	0.487 [0.264-0.896]	0.021
Yes (n = 119)	0.824	0.148	0.028	0.100			

Inheritance models were adjusted for age, gender, and BMI. >p75: >75th percentile; <p25: <25th percentile.

**Table 6:** Association between *PCSK9* gene polymorphisms and metabolic abnormalities in the control group.

Polymorphism	Genotype Frequency			MAF	Model	OR [95% CI]	*p*
**rs2479409**	***GG***	***GA***	***AA***				
Metabolic syndrome							
No (n = 678)	0.445	0.441	0.014	0.334	Dominant	0.768 [0.593–0.994]	0.045
Yes (n = 424)	0.531	0.382	0.087	0.278			
Alkaline phosphatase >p75							
No (n = 692)	0.443	0.440	0.118	0.338	Additive	0.758 [0.625–0.919]	0.005
Yes (n = 410)	0.538	0.381	0.081	0.271	Dominant	0.703 [0.548–0.903]	0.006
					Codominant 1	0.729 [0.560–0.950]	0.019
					Codominant 2	0.603 [0.386–0.943]	0.027
Gamma-glutamyltranspeptidase >p75							
No (n = 649)	0.445	0.450	0.105	0.330	Heterozygote	0.760 [0.588–0.982]	0.036
Yes (n = 453)	0.525	0.373	0.102	0.288	Codominant 1	0.751 [0.575–0.981]	0.036
							
**rs615563**	***GG***	***GA***	***AA***				
Hypertriglyceridemia							
No (n = 580)	0.722	0.238	0.040	0.160	Recessive	0.358 [0.149–0.862]	0.022
Yes (n = 522)	0.728	0.259	0.013	0.143	Codominant 2	0.374 [0.155–0.903]	0.029
Interleukin 1β > p75							
No (n = 681)	0.700	0.265	0.036	0.167	Additive	0.767 [0.598–0.985]	0.037
Yes (n = 421)	0.752	0.231	0.018	0.134			

Inheritance models were adjusted for age, gender, and BMI. >p75: >75th percentile.

## Data Availability

The data shown in this article are available upon request from the corresponding author.
